# Supplementary material for: Coordinated representational reinstatement in the human hippocampus and lateral temporal cortex during episodic memory retrieval
Source: Nat Commun. 2019 May 21;10:2255. doi: 10.1038/s41467-019-09569-0 (PMC6529470; doi:10.1038/s41467-019-09569-0)
Supplement: Supplementary file 1 — Supplementary Information [file 41467_2019_9569_MOESM1_ESM.pdf]

SUPPLEMENTARY INFORMATION

Coordinated representational reinstatement in the human hippocampus and lateral temporal cortex during episodic memory retrieval

Pacheco et al., *Nat. Commun.,* 2019

SUPPLEMENTARY NOTES

Supplementary Note 1: Analysis of hits versus correct rejections

In the hits versus correct rejection analysis, we observed a significant cluster of increased ERS ranging from 2 to 2.5s in encoding time and from 1.3 to 2.2s in retrieval time (p = 0.04; see top row in Supplementary Fig. 13). These time windows overlap substantially with cluster ii from our original analysis. In the high confidence hits versus high confidence correct rejection analysis, an even more extended cluster of reinstatement was observed, ranging from 1.5 to 3.5s at retrieval and from 2 to 3.5s at encoding (p = 0.01; see bottom row in Supplementary Fig. 13). Again, no item-specific activity was observed in the hippocampus (hits versus correct rejections: all $p > 0.184$; high confidence hits versus high confidence correct rejections: all $p > 0.209$), further confirming our initial results (Supplementary Fig. 14). Taken together, these results confirm the distinct and content-specific forms of reinstatement we observed in the Hippocampus and the LTC in the main analysis.

Supplementary Note 2: Interaction analysis

To check for a possible interaction of context and item reinstatement in the hippocampus and the LTC, we compared reinstatement for hits versus correct rejections between congruent and incongruent spatial context conditions in these two regions. We first subtracted hits and correct rejections for congruent and incongruent trials separately, and then statistically compared these contrast values. This was necessary because applying the surrogate-based cluster analysis to interactions (in this case: hits vs. correct rejection x congruent vs. incongruent) requires first computing contrasts in individual conditions, and then analyzing the interaction effect as the difference between the contrasts. Results

of this interaction analysis in the hippocampus revealed a significant cluster in a very similar time window as the cluster observed in the original congruency contrast (*p* = 0.014; Supplementary Fig. 15). This result clearly supports our main conclusion that hippocampal reinstatement – now measured via the contrast of reinstatement during hits vs. correct rejections – is context-dependent. In the LTC, on the other hand, no significant cluster was observed (all $p > 0.206$; Supplementary Fig. 16). In addition to our previous analyses, this result underlines that reinstatement is context-dependent in the hippocampus but not in the LTC.

Supplementary Note 3: Comparison of correct and incorrect trials

We directly compared reinstatement in correct and incorrect trials, despite the low number of incorrect trials that were captured (only in 7 of 11 subjects; trial numbers: 3, 21, 16, 6, 4, 2, 2). In the LTC we observed that, numerically, ERS was higher for correct vs. incorrect trials around the same temporal region of interest where we observed a confidence effect before (even though no cluster survived correction for multiple comparisons; see Supplementary Fig. 12). When we restricted analysis to the cluster where a confidence effect had been observed, mean ERS for correct items was significantly greater than zero ($t(10) = 3.59$, $p = 0.004$), while it did not differ from zero in the incorrect trials ($t(6) = -0.661$, $p = 0.533$).

Similarly, when we focused our analysis on the LTC reinstatement cluster ii (resulting from the orthogonal contrast of same vs. different items during encoding and retrieval) in a similar time period, we found that reinstatement values were significantly greater than zero for correct trials ($t(10) = 5.395$; $p = 0.0003$), but not for incorrect trials ($t(6) = -0.401$, $p = 0.701$).

Supplementary Note 4: Analysis of reinstatement at increased temporal resolution

Hippocampal reinstatement of item-context associations occurred very early after the onset of item presentations during retrieval (0-500ms). This can be explained by several factors. First, in the experimental paradigm, participants have already entered a specific room before any item is presented during retrieval – they have to reach the end of the rooms before being presented with items, which takes about 500-1000ms depending on navigation speed. Thus, contextual information is already available to them when retrieval starts. It is therefore likely that a contextual signal that is present before item onset modulates hippocampal ERS. Importantly, we note that this contextual signal alone cannot explain the effect of congruency on hippocampal ERS, as demonstrated in the contrast of same vs. different rooms (independent of items; see Figure 2C).

Second, please note that we plot the results corresponding to a specific time window at the onset of individual time bins (see Methods). That is, activity marked at zero in the reinstatement maps includes signals ranging from 0 to 500ms, activity marked at 100ms includes activity between 100-600ms after onset etc. Given that the final calculation of ERS is applied to an entire 500ms time window, the apparent early onset may be due to increases in ERS occurring hundreds of milliseconds later.

In order to address the timing of ERS effects more specifically, we performed an additional analysis in which we increased the temporal resolution by a factor of 10, i.e., we constructed representational patterns in windows of 50ms, sliding with a 10ms increment. We specifically focused on temporal windows of 1.5s duration centered around the effects that were observed previously (hippocampus: 1.5-3s in encoding time, -0.5-1s at retrieval; LTC: 1-2.5s at encoding, 2-3.5s at retrieval). These analyses revealed increases in hippocampal ERS after about 400ms (p = 0.026, Supplementary Fig. 17, top). The onset of effects in the LTC, identified in the confidence contrast, were observed

starting at ~1.5s after cue presentation and lasted until 1.9s, i.e., until the time before participants gave their responses (p = 0.018; Supplementary Fig. 17, bottom).

Supplementary Note 5: Control analyses in HC (posterior and multi-electrode)

To assess the spatial specificity of our results in the anterior hippocampus and the LTC, we conducted the following additional analyses: (1) we calculated RSA at a posterior hippocampal contact in each patient; (2) we calculated hippocampal RSA including activity from all available hippocampal contacts; (3) we built LTC RSA patterns from the activity of all available LTC contacts (see Methods and Supplementary Data 1 for details). In addition, we analyzed reinstatement in the parietal cortex as a control region.

These complementary analyses revealed that hippocampal reinstatement is mostly driven by its anterior part. Indeed, no clusters survived multiple comparisons correction in the posterior hippocampal analysis or in the analysis including all hippocampal contacts (all $p > 0.180$; Supplementary Fig. 4, panels A and D). However, when focusing on the previously identified temporal hippocampal cluster, we found a significant difference between congruent and incongruent trials in the multi-electrode analysis ($t(7) = 2.397$; $p = 0.048$; Supplementary Fig. 4, E). While this difference was not observed in the posterior hippocampus, ERS was significantly higher than zero also in that region for congruent but not for incongruent trials (congruent: $t(5) = 2.798$; $p = 0.038$; incongruent: $t(5) = 0.397$; $p = 0.707$; Supplementary Fig. 4, B).

In the LTC, results of the multi-electrode analysis revealed no item or context-specific reinstatement (all $p > 0.109$), suggesting a localized representation of items in more anterior regions of the temporal lobe where the electrodes of the main analysis are located. In the confidence contrast, we observed a significant ERS increase for high as compared

to low confidence trials starting 2 seconds after cue onset in encoding and 3 seconds in retrieval time (p = 0.035; Supplementary Fig. 5).

In the parietal cortex, we did not find any difference between conditions in the context (all $p > 0.110$) or item comparisons (all $p > 0.129$). However, we observed increased reinstatement for high versus low confidence trials around 2 seconds after cue presentation in encoding and retrieval time (p = 0.0108), suggesting an involvement of the parietal cortex in the encoding of subjective confidence (Supplementary Fig. 6).

Supplementary Note 6: Analysis of lateralization effects

In order to specifically test possible influences of lateralization, we assessed reinstatement in our main contrasts by only including patients with either left or right implantations. In the hippocampus (n=5 left, n=3 right), we did not find significant differences between congruent and incongruent items in either the left (all $p > 0.258$) or the right hemisphere group (all $p > 0.07$; Supplementary Fig. 7). In the LTC (n=7 left, n=4 right), item-specific effects were observed in the left hemisphere around the time of cluster ii in the main analysis (p = 0.02). In the right hemisphere group, no differences were observed between conditions (all $p > 0.288$; Supplementary Fig. 8).

In addition, we directly compared reinstatement patterns between patients with electrodes in the left and right hemisphere. For this analysis, we focused on contrast values in the previously identified clusters. Mean ERS values in the hippocampal congruent-incongruent cluster were not statistically different between hemispheres ($t(7) = -0.31$, p = 0.761, unpaired t-test; Supplementary Fig. 7). In the LTC, we compared mean ERS values between left and right hemisphere groups in cluster ii of the main analysis (i.e., where item and confidence effects were found). Again, no statistical difference was

observed ($t(9) = 0.044$, $p = 0.965$, unpaired t-test; Supplementary Fig. 8).

Supplementary Note 7: Coordinated reinstatement analysis

Since our aim in the coordinated reinstatement analysis was to investigate the interaction of the different representational formats of reinstatement in the hippocampus and the LTC, we defined temporal regions of interest in each region that were aligned in time during encoding. Specifically, we only included in our analysis those encoding/retrieval time pairs that satisfied two conditions: First, significance in each region's contrast; and second, identical encoding time. The rationale behind this approach was that coordinated reinstatement reflects activity from the same encoding period. Thus, we specifically investigated coordinated reinstatement during the time window in which we had observed content-specific reinstatement in the two individual regions of interest. In order to further validate the specificity in time of the HC/LTC interaction, we also analyzed coordinated reinstatement across all possible encoding/retrieval time windows. This analysis showed that coordinated reinstatement across regions is indeed specific to those time windows where reinstatement in the two individual regions occurs (Figure 5).

Please note that these correlations were obtained across trials within individual participants. In principle, it would also have been interesting to correlate the magnitude of the respective ERS contrasts in HC and LTC. However, such a correlation of contrast values would only have been possible across participants, which is generally a less direct approach because inter-individual differences are driven by various different factors including age, gender, cognitive status etc.; and, in the case of patient groups: pathology. For these reasons we have not pursued that possibility.

Supplementary Note 8: LTC results in patients without hippocampal epilepsy

To confirm that our results in the LTC were not affected by hippocampal epileptic activity (diagnosed in three subjects that were included in our main analyses, Figure 3), we performed all analyses involving the LTC in a smaller group of patients excluding those with hippocampal epilepsy. We observed qualitatively the same results as is the main analysis. Two clusters of item-specific reinstatement were observed in the same positions in time (cluster i: $p_{(corr)} = 0.030$, cluster ii: $p_{(corr)} = 0.032$; see Supplementary Fig. 19A). Average ERS in the "same item" condition was significantly higher than zero in both clusters (cluster i: $t(7) = 5.353$, $p = 0.001$; cluster ii: $t(7) = 2.855$, $p = 0.024$), while it did not differ from zero in the "different item" condition (cluster i: $t(7) = 1.327$, $p = 0.225$; cluster ii: $t(7) = 0.576$, $p = 0.582$ Supplementary Fig. 19B). We did not observe any reinstatement of item-context associations (all clusters, $p > 0.495$; Supplementary Fig. 19C), or room-specific activity (all clusters, $p > 0.346$; Supplementary Fig. 19D). We also found a cluster of significant ERS increases in the high versus low confidence comparison ($p_{(corr)} = 0.038$, Supplementary Fig. 19E). In this cluster, reinstatement of high-confidence trials was significantly larger than zero ($t(6) = 3.757$, $p = 0.009$), while it did not differ from zero for low-confidence trials ($t(6) = -1.699$, $p = 0.140$; Supplementary Fig. 19F). Please note that the cluster identified in this latter confidence contrast was used in all analyses involving the interaction of the hippocampus and the LTC (Methods).

Supplementary Note 9: Response-locked analysis

Previous studies (e.g., see ref. [1]) observed significant condition effects of response-locked reinstatement which did not occur in our data (hippocampal congruent-incongruent contrast: all $p > 0.433$; LTC item contrast: all $p > 0.373$; see Supplementary Fig. 18). In general, our paradigm is less suited for detecting response-locked ERS increases than

previous studies. This is because: (1) we did not ask participants to respond as fast as possible, and thus various factors may account for the variance of responses across trials – factors that are independent of the trial-specific timing of reinstatement; (2) additional variance of response times is added by the fact that the memory test in our experiment is integrated into a spatial navigation paradigm. Note that ref. [1] also used an associative memory test, and the mean reaction time they observed is similar to ours (1831 vs. 1872ms for correct trials; 2736 vs. 2617ms for incorrect trials). However, the variability (STD) reported in their analyses is considerably smaller for both correct and incorrect trials (correct: 452 vs. 625ms; incorrect: 656 vs. 925ms). The relatively high variability in the response times of our subjects might explain the lack of significant effects we observed in the response-locked analysis. (Note that we converted variability from SEM as reported in their paper to STD in order to allow for a meaningful comparison with our results given the different numbers of subjects.)

Supplementary Note 10: Coordinated reinstatement between HC and PPC

In order to investigate whether our results in the coordinated reinstatement analysis (Figure 5) are specific to the relationship of the anterior hippocampus with the lateral temporal cortex, we calculated "coordinated reinstatement" between the hippocampus and the posterior parietal cortex (PPC) as a control. As in the HC-LTC interaction analysis, we correlated single trials levels of hippocampal congruent-incongruent reinstatement and PPC confidence reinstatement in their respective temporal regions of interest (hippocampus: congruent incongruent cluster, PPC: confidence cluster). In the ERS same item, all contexts condition, we found a non-significant relationship ($t(4)= 0.135$; $p = 0.89$, see Supplementary Fig. 20), suggesting that the hippocampus specifically coordinates with the LTC during the retrieval of episodic memories. However, we note

that the relatively low number of subjects with parietal electrodes and the higher variance in the spatial position of parietal contacts as compared to the LTC (see Supplementary Figs. 5 and 6) might have affected these results. In addition, we stress the need of further exploring the coordination of different forms of reinstatement across the brain (e.g., between the hippocampus and the prefrontal cortex, given the well-known involvement of the latter in memory retrieval during systems consolidation; see ref. [2]).

SUPPLEMENTARY FIGURES

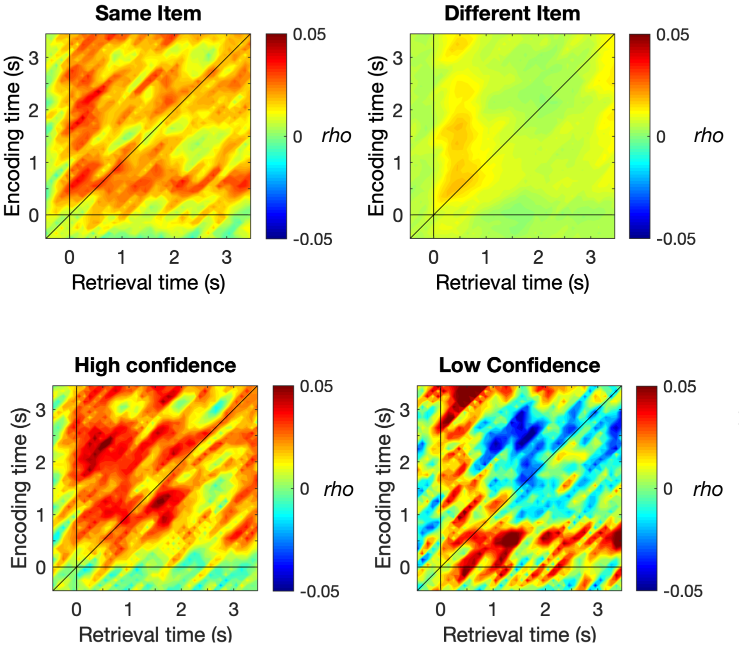

Supplementary Figure 1: Lateral temporal cortex reinstatement maps

Average reinstatement in the lateral temporal cortex for same, different, high and low confidence conditions. Areas of significant differences between conditions are presented in Figure 3 and described in the main text.

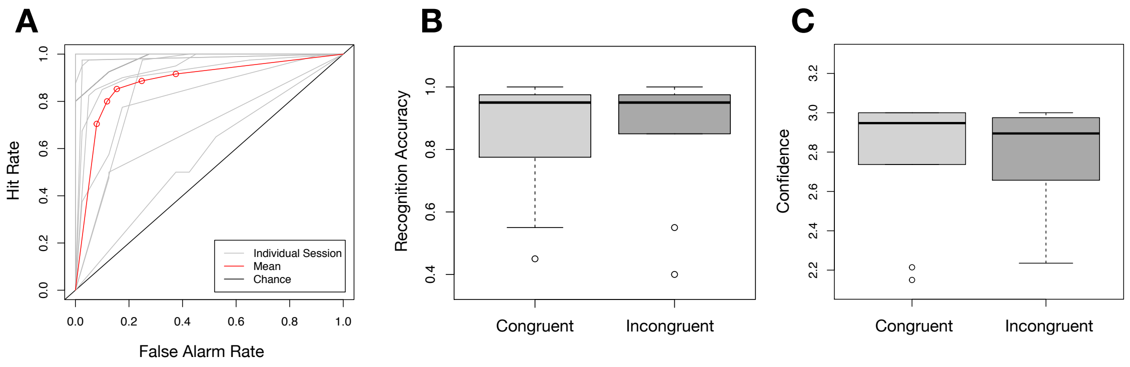

Supplementary Figure 2: Behavioral Results

(A) Receiver Operating Characteristic (ROC) curve for individual subjects (N = 11, grey) and group average (red). Each data point corresponds to a different confidence level. **(B)** Recognition accuracy (% correct) for congruent and incongruent trials. **(C)** Retrieval confidence in congruent and incongruent trials.

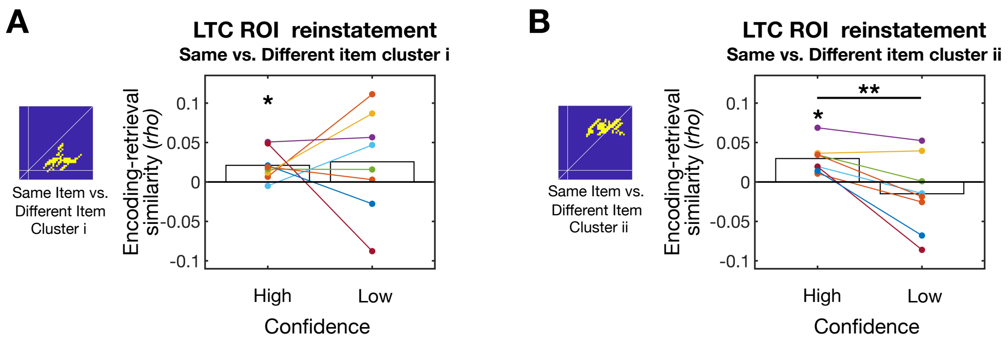

Supplementary Figure 3: Item-specific and confidence reinstatement in the LTC

Mean ERS for high and low confidence trials in cluster i (A) and ii (B) of the lateral temporal cortex item specific contrast. * and ** indicates *p*-values below 0.05 and 0.01 respectively (one sample *t*-test in the comparison against zero in panels A and B, and paired *t*-test for the comparison between conditions in panel B).

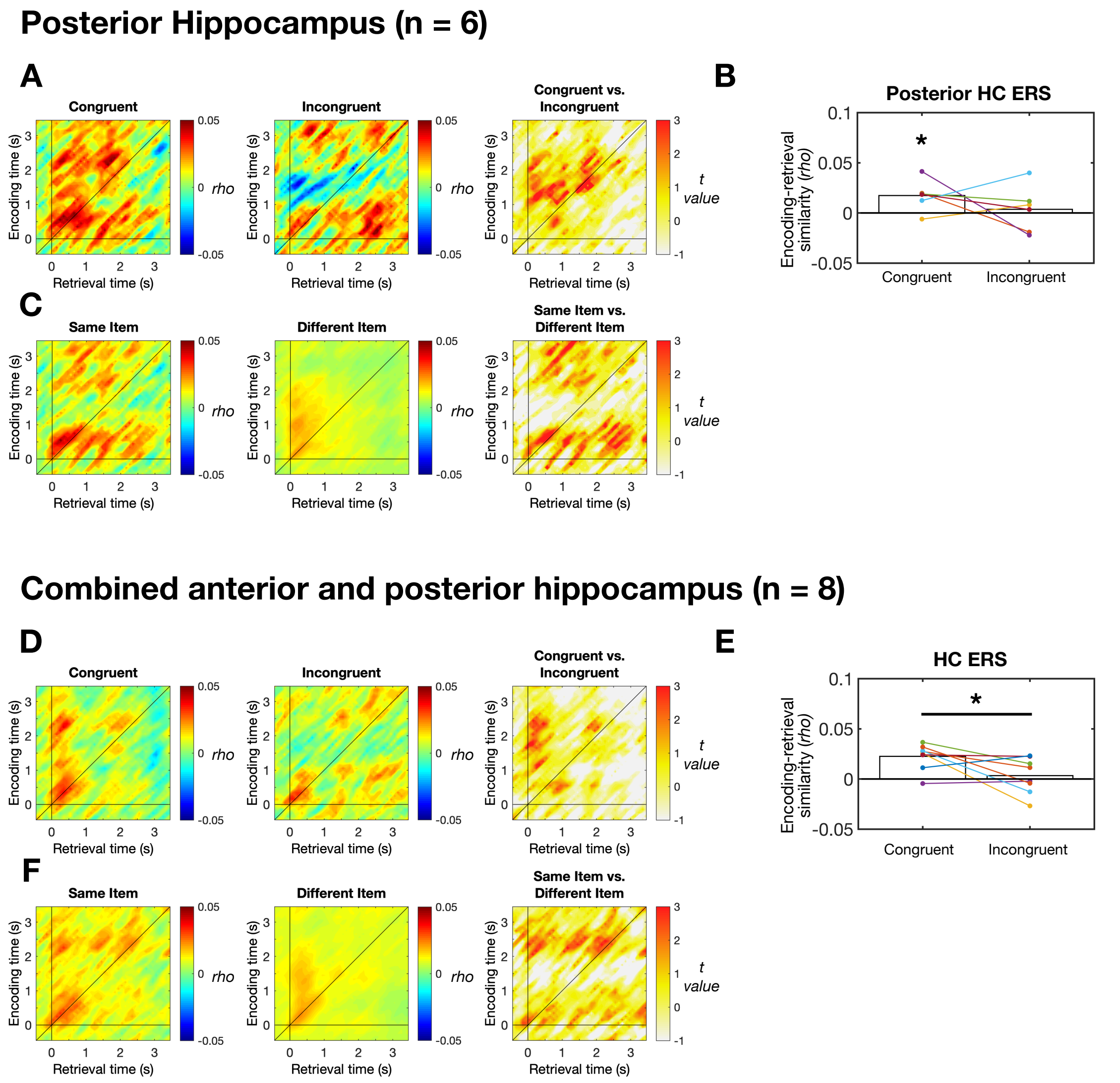

Supplementary Figure 4: ERS in posterior and in combined anterior-posterior HC

(A) Lack of congruency effect in posterior hippocampus (B) Analysis of congruency effects in previously identified cluster from the anterior hippocampus showing significant encoding-retrieval similarity for congruent but not for incongruent items. (C) Lack of item-specific reinstatement in the posterior hippocampus. (D) Lack of congruency effect in the multi-electrode analysis. (E) Analysis of congruency effects in previously identified cluster from the anterior hippocampus, now including concatenated activity from all available hippocampal contacts. Plot shows significantly higher ERS for congruent as compared to incongruent items. * indicates a *p*-value below 0.05 (paired *t*-test). (F) Lack of item-specific reinstatement in the multielectrode analysis.

Lateral Temporal Cortex global reinstatement analysis (n = 11)

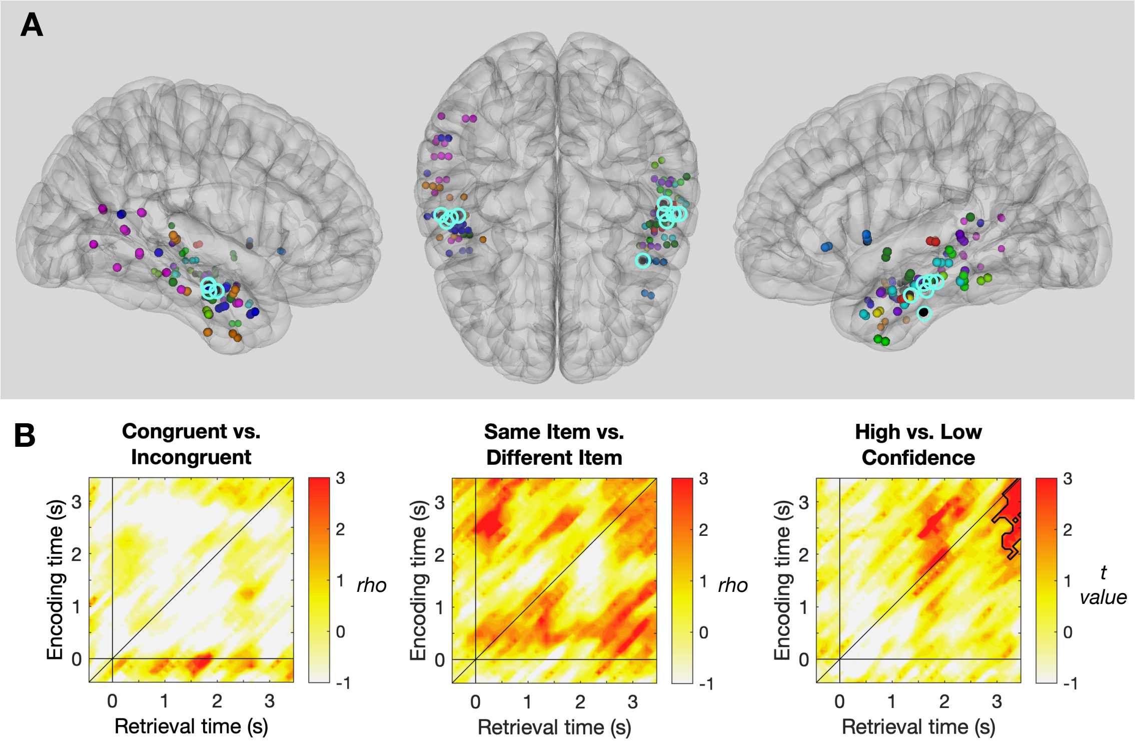

Supplementary Figure 5: LTC reinstatement based on multi-electrode patterns

(A) All available electrodes in the LTC of each patient are plotted in common MNI space (see also Supplementary Data 1). Cyan circles indicate locations of electrodes that were chosen in each subject for the single-electrode LTC analysis. (B) Results of the multi-electrode ERS analysis showing significant reinstatement for high versus low confidence trials (right); lack of significant congruency (left) or item-specific effects (middle).

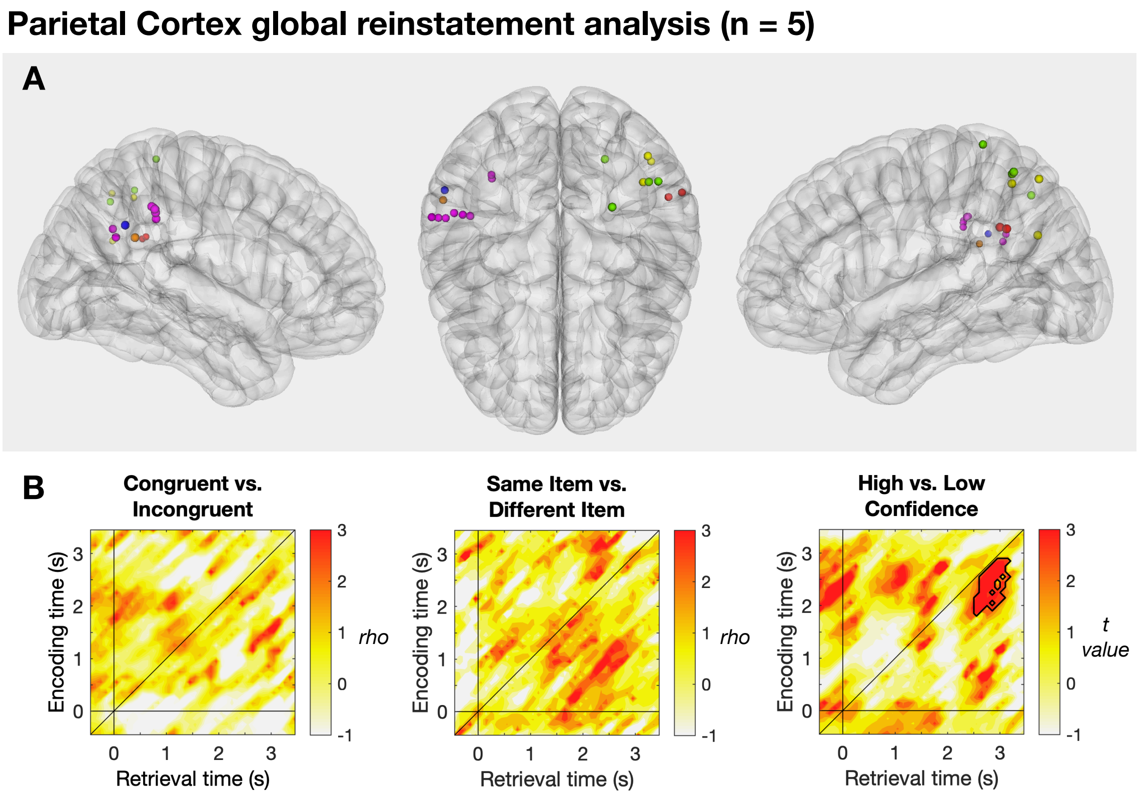

Supplementary Figure 6: Reinstatement effects in the posterior parietal cortex

(A) Global RSA analysis was performed with concatenated activity from all available parietal electrode contacts in each patient, here shown in common MNI space (see also Supplementary Data 1). Each color represents a different subject. (B) Lack of reinstatement of item-context associations (left), or item-specific information (middle). Right: Significantly higher ERS for items rated with high as compared to low confidence.

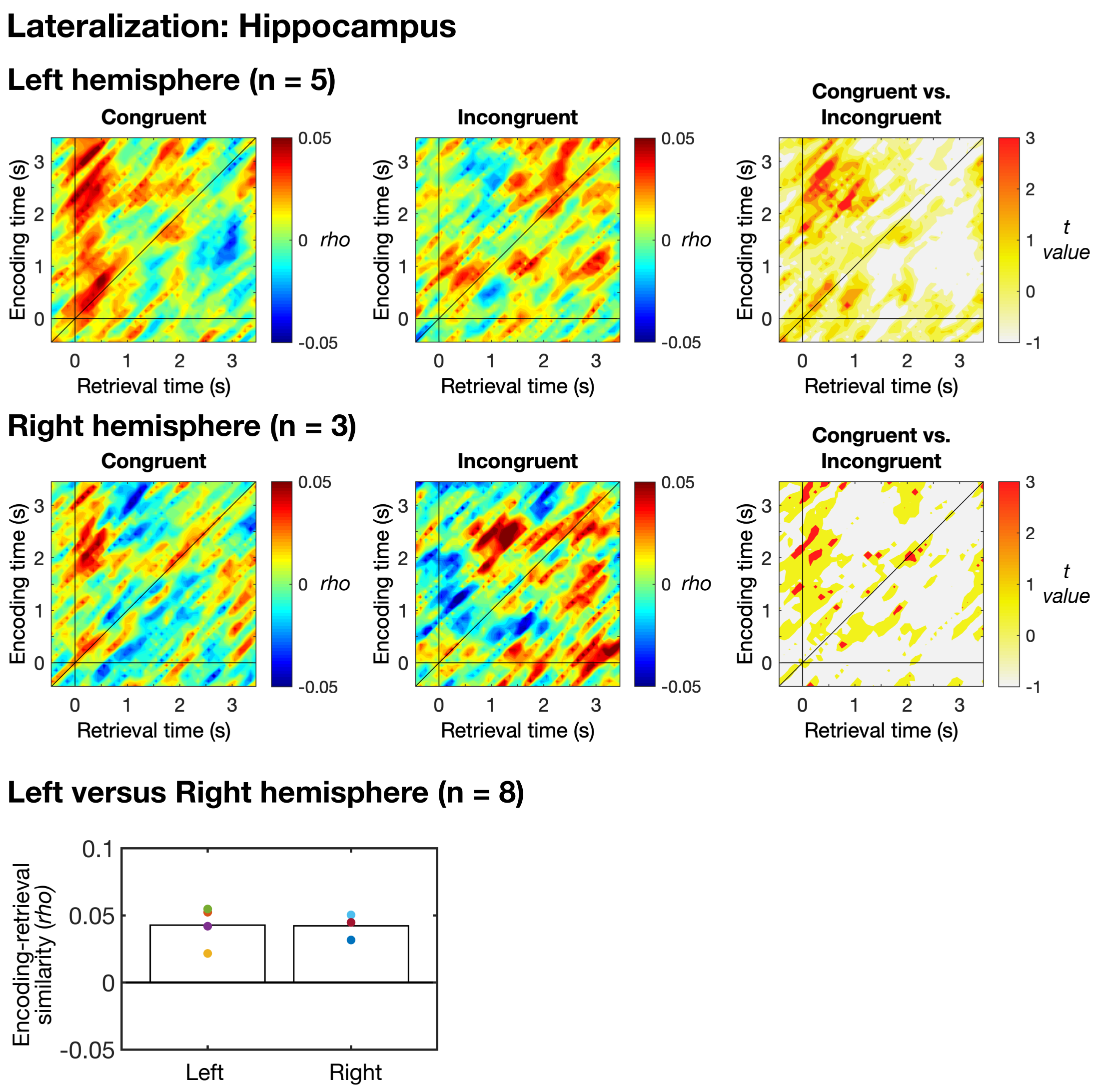

Supplementary Figure 7: HC reinstatement in left and right hemispheres

Top and middle rows: no significant reinstatement was observed when analyzing the two patient groups separately. Bottom row: a direct comparison of mean ERS values within the hippocampal congruent – incongruent cluster revealed no statistical differences between the two groups.

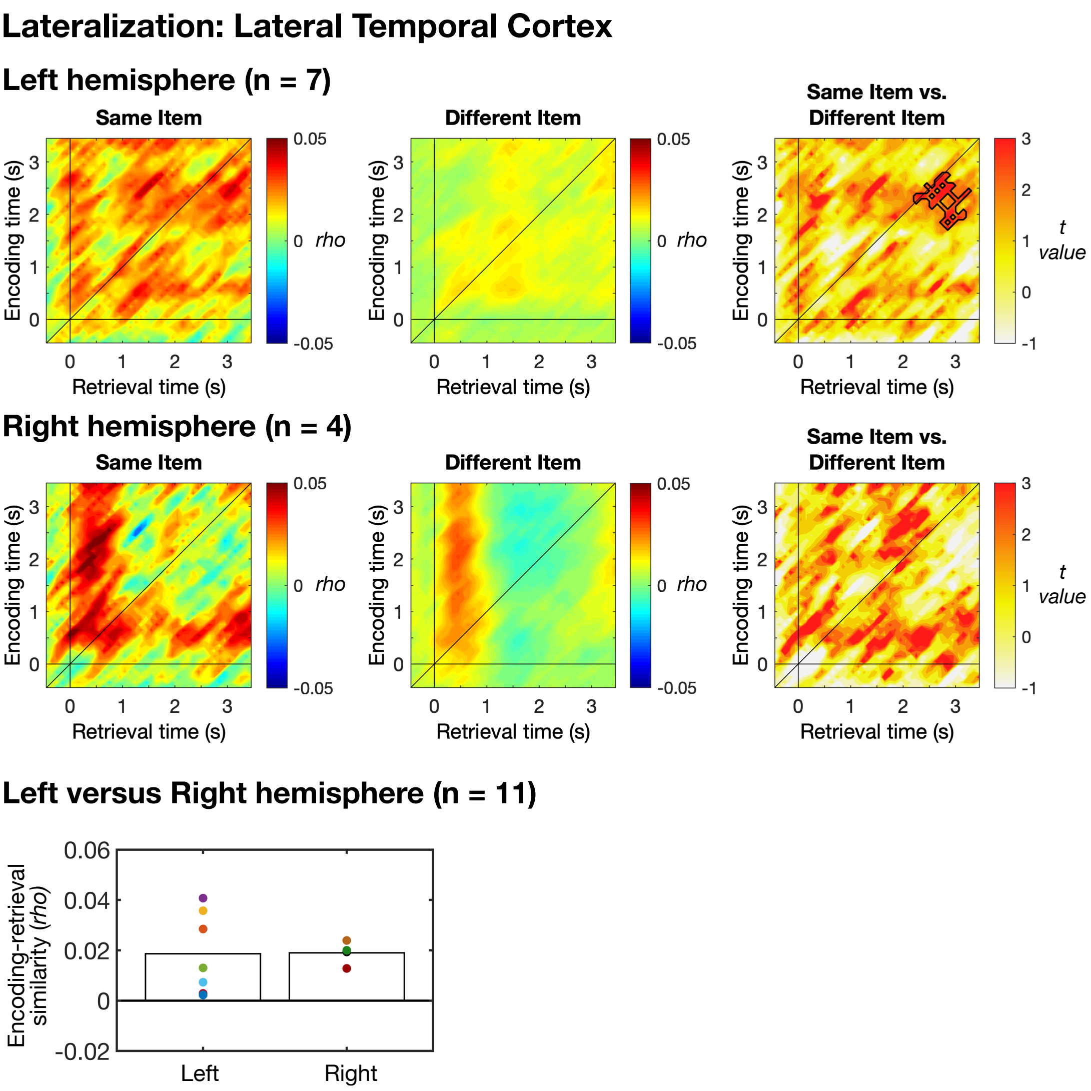

Supplementary Figure 8: LTC reinstatement in left and right hemispheres

Top row: significant item-specific reinstatement was observed in the left hemisphere group in the LTC, while this effect was not present in the right hemisphere group (middle). Bottom row: a direct comparison of mean ERS values within the item-specific cluster (nº ii of the main analysis) revealed no statistical differences between the two groups.

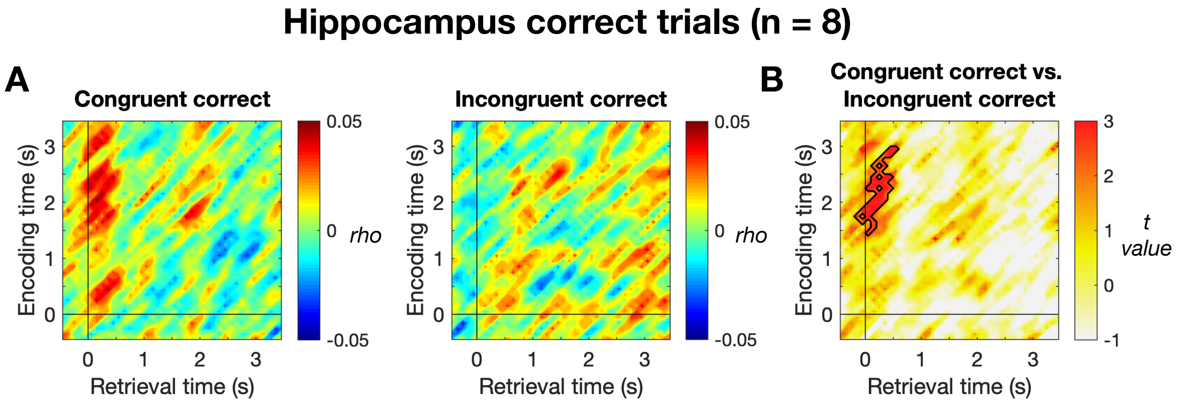

Supplementary Figure 9: Congruency effects for correct trials in the hippocampus

A significant cluster was observed at a very similar point in time as in the main analysis (congruent-incongruent hippocampal cluster: p = 0.019)

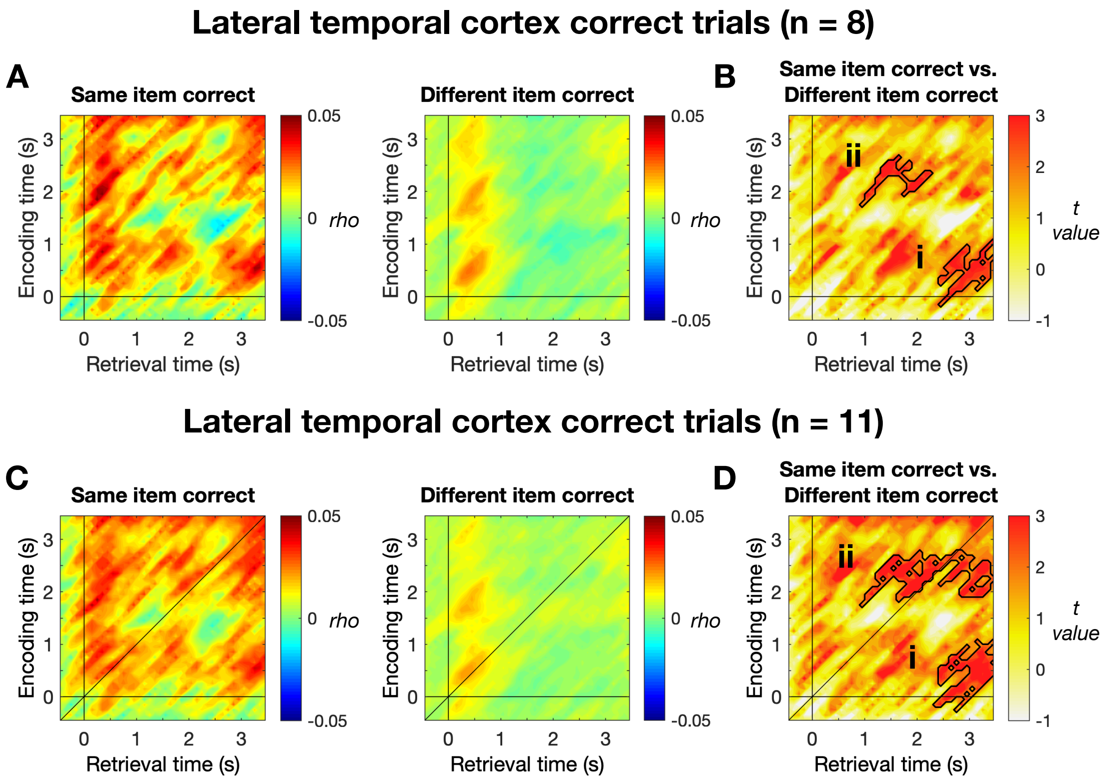

Supplementary Figure 10: LTC item-specific reinstatement in correct trials

(A,B): Reinstatement effects in n=8 patients without hippocampal epilepsy. Two significant clusters were observed as in the main analysis (cluster i: p = 0.027; cluster ii: p = 0.044). (C,D): Reinstatement effects in the extended group of n=11 patients. Again, two significant clusters were observed (cluster i: p = 0.018; cluster ii: p = 0.011).

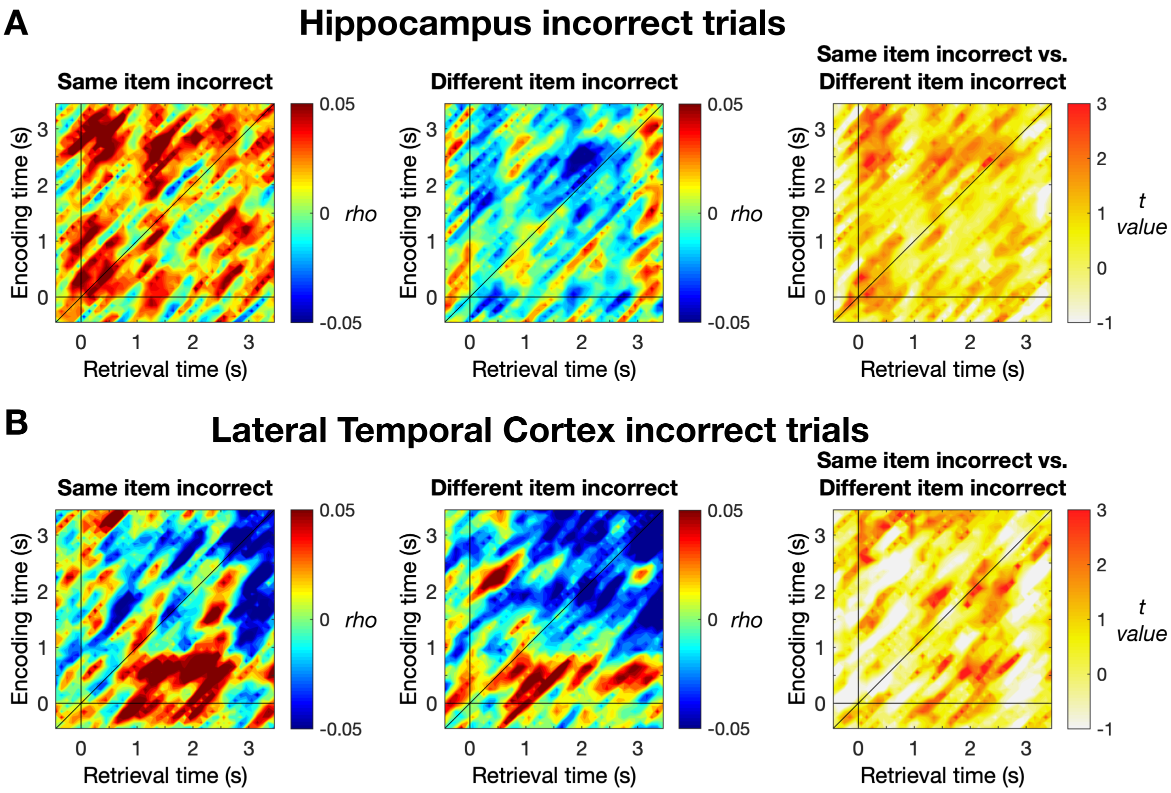

Supplementary Figure 11: Item reinstatement in incorrect trials in HC and LTC

Lack of reinstatement of item-specific activity during incorrect trials in the hippocampus (all p > 0.272, A) and the lateral temporal cortex (all p > 0.159; B).

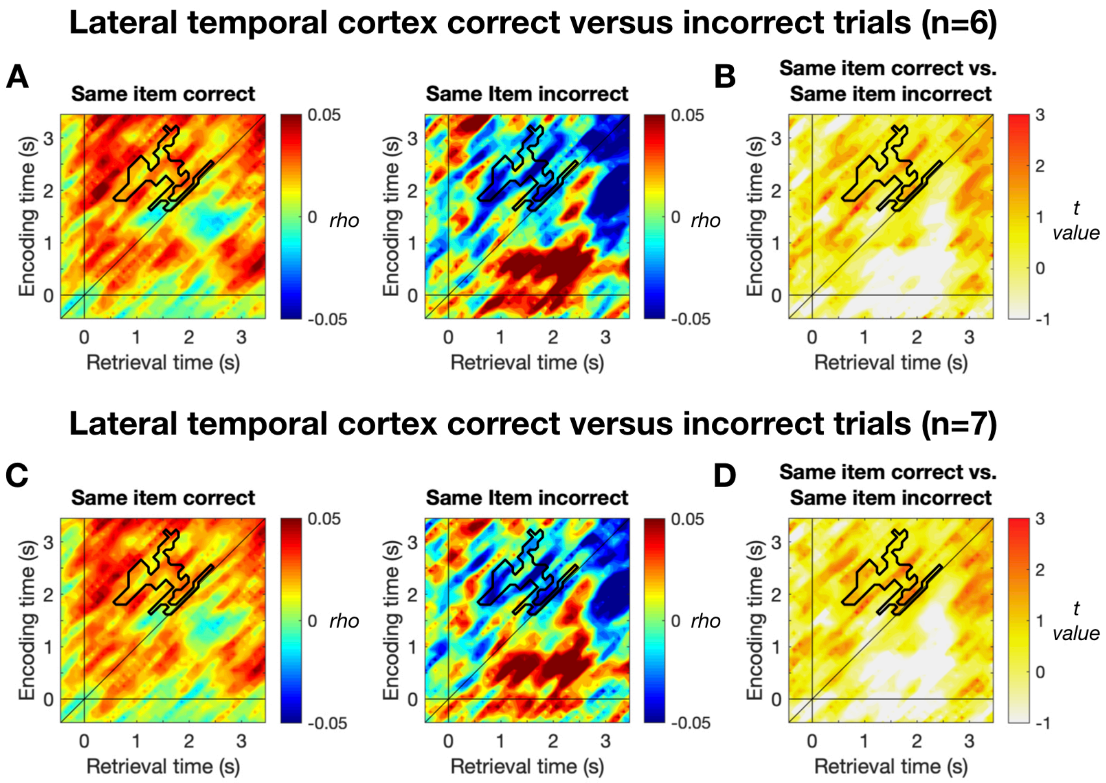

Supplementary Figure 12: Reinstatement for correct and incorrect trials in LTC

(A,B): Patients without hippocampal epilepsy. (C,D): Extended group of patients.

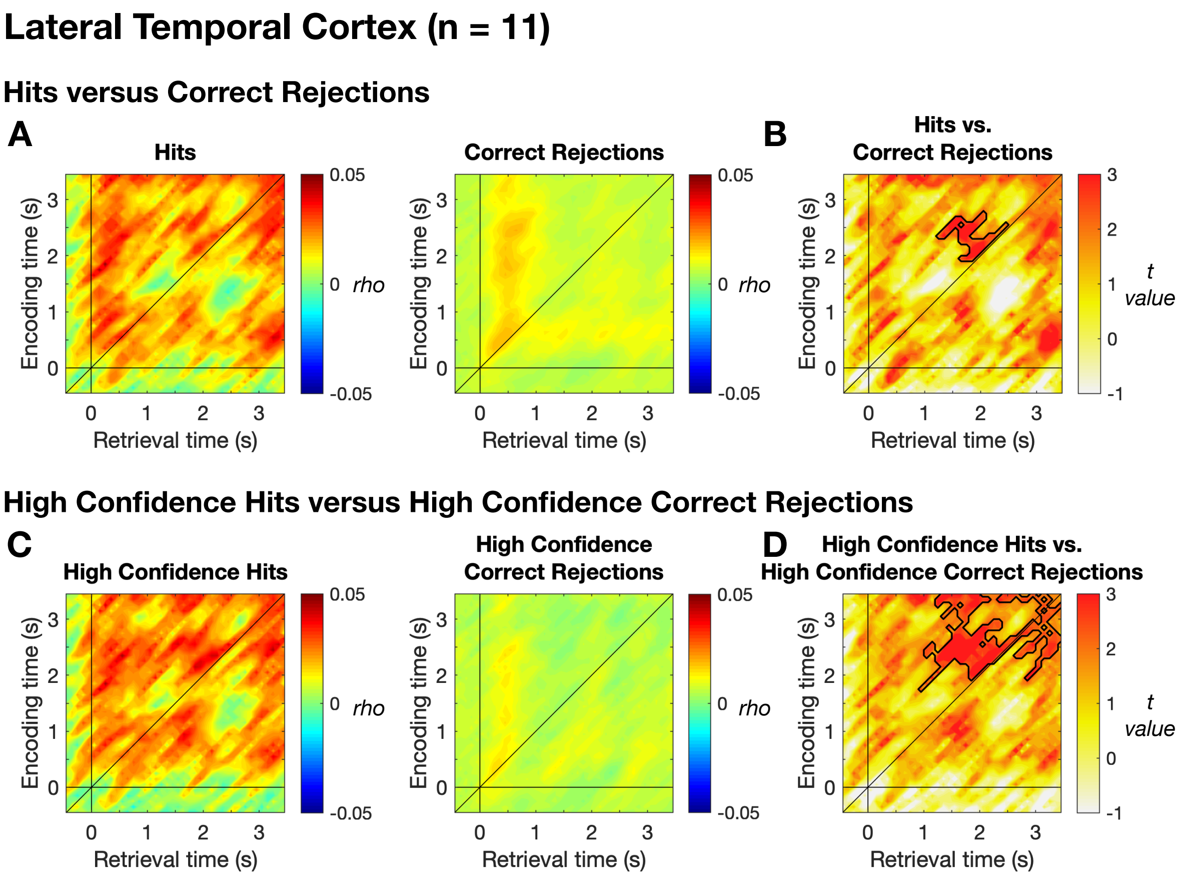

Supplementary Figure 13: Reinstatement for hits and correct rejections in the LTC

(A) Reinstatement maps for hits and correct rejections in the lateral temporal cortex (B) *T*-map resulting from the statistical comparison of maps in panel A. (C) Reinstatement maps for high confidence hits and high confidence correct rejections in the lateral temporal cortex (D) *T*-map resulting from the statistical comparison of maps in panel C.

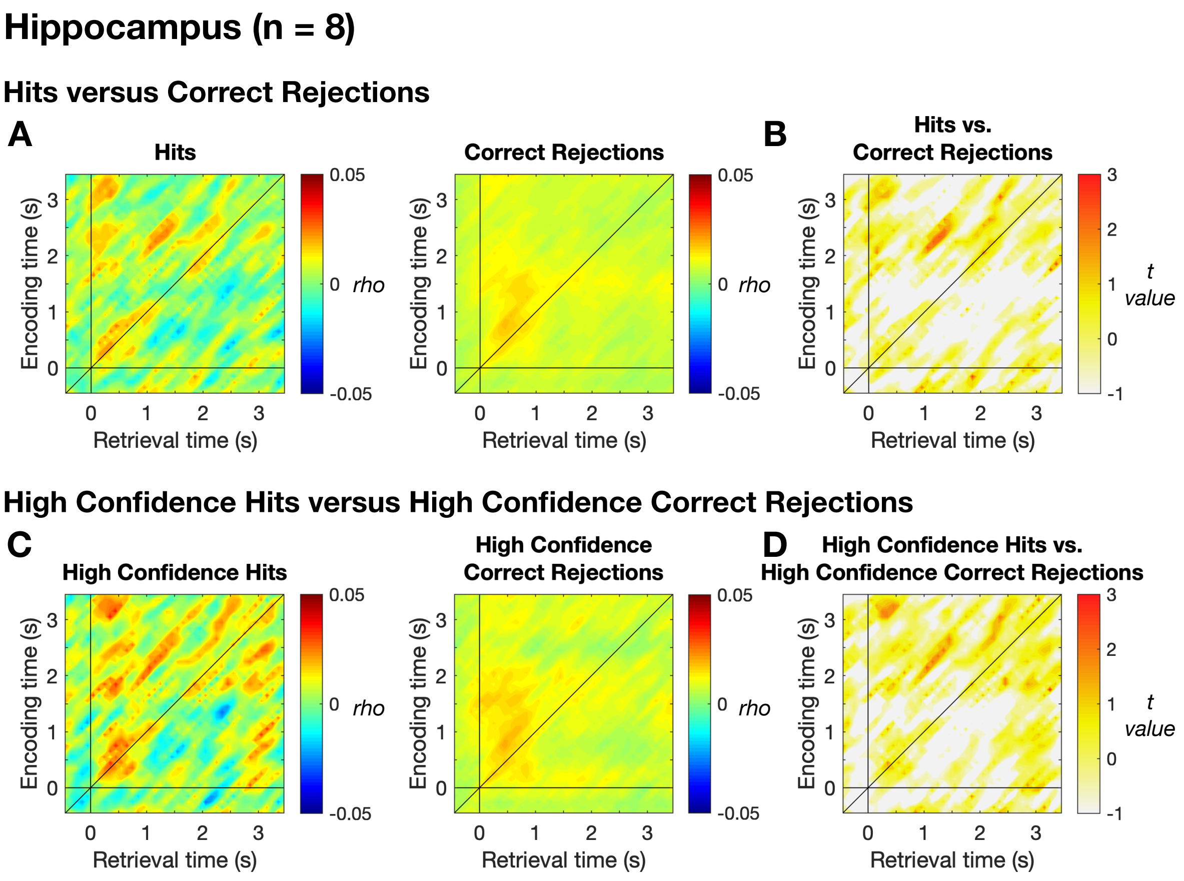

Supplementary Figure 14: Reinstatement for hits and correct rejections in the HC

(A) Reinstatement maps for hits and correct rejections in the hippocampus (B) *T*-map resulting from the statistical comparison of maps in panel A. (C) Reinstatement maps for high confidence hits and high confidence correct rejections in the hippocampus (D) *T*-map resulting from the statistical comparison of maps in panel C.

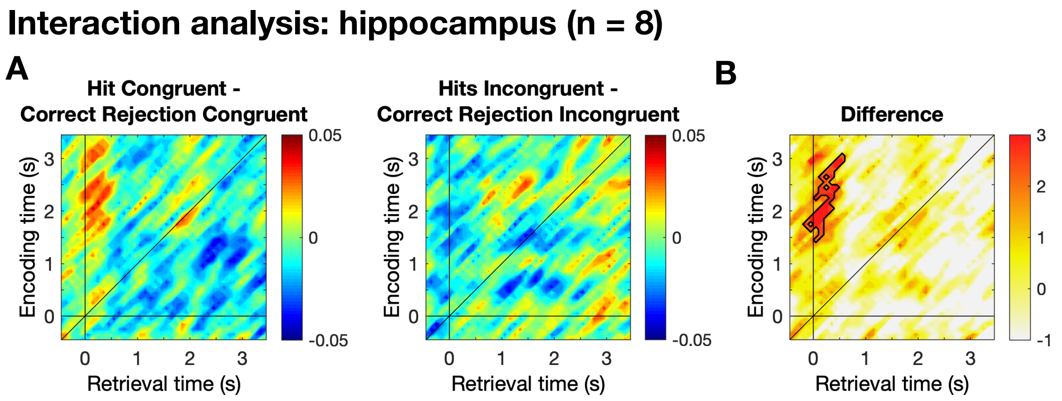

Supplementary Figure 15: Interaction analysis in the hippocampus

(A) Reinstatement maps for hits minus correct rejections for congruent (left) and incongruent (right) conditions in the hippocampus. (B) *T*-map resulting from the statistical comparison of maps in panel A.

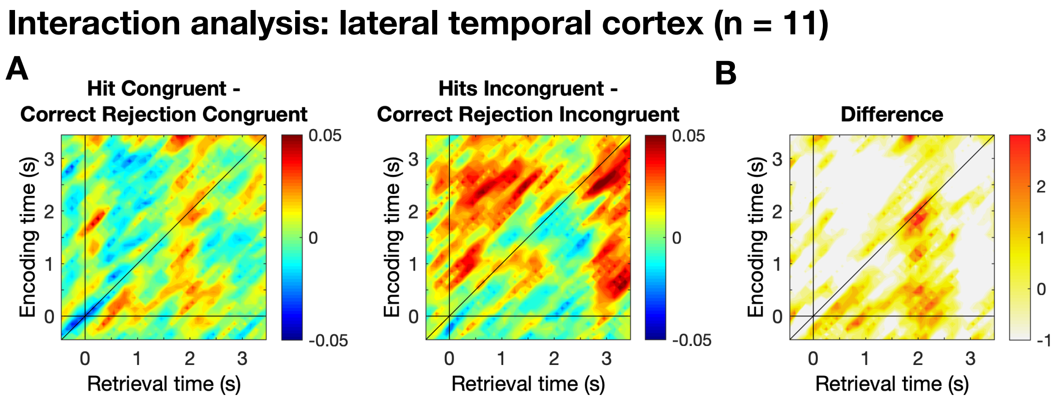

Supplementary Figure 16: Interaction analysis in the LTC

(A) Reinstatement maps for hits minus correct rejections for congruent (left) and incongruent (right) conditions in the lateral temporal cortex. (B) *T*-map resulting from the statistical comparison of maps in panel A.

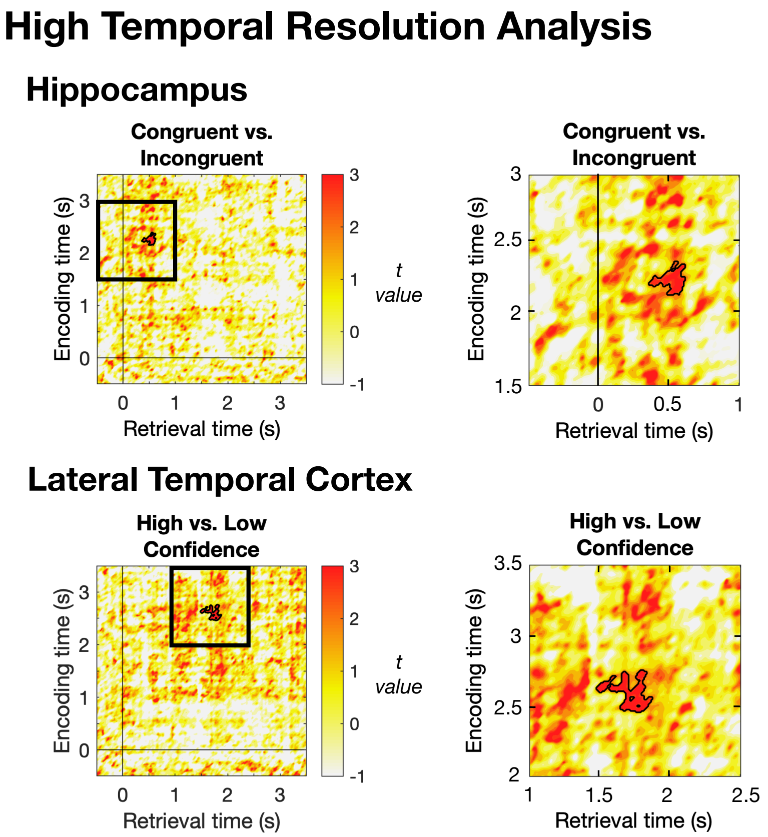

Supplementary Figure 17: Reinstatement effects at higher temporal resolution

Top row: hippocampal reinstatement of item-context associations. Bottom row: lateral temporal cortex reinstatement of item-specific information.

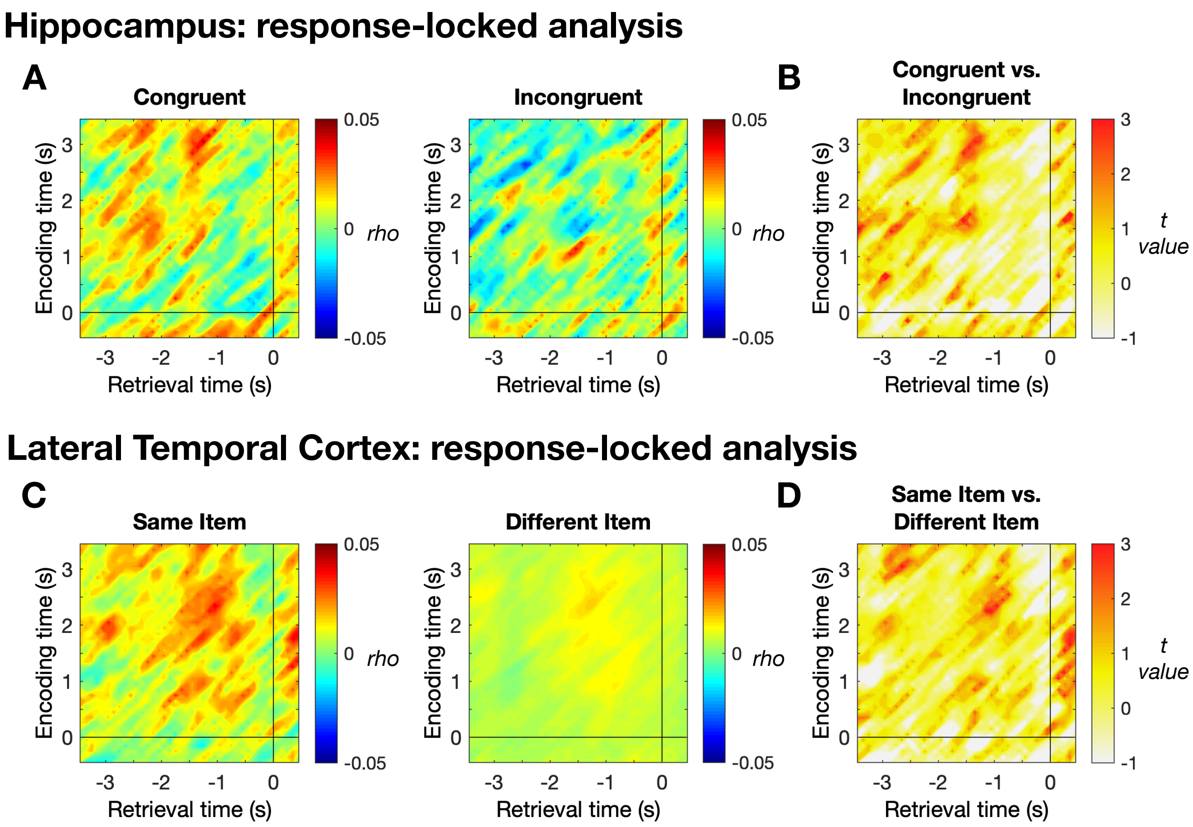

Supplementary Figure 18: Response-locked analyses

(A,B): Lack of context effects in the hippocampus in the response-locked analysis. (C,D): Lack of item-specific reinstatement in the lateral temporal cortex in the response-locked analysis.

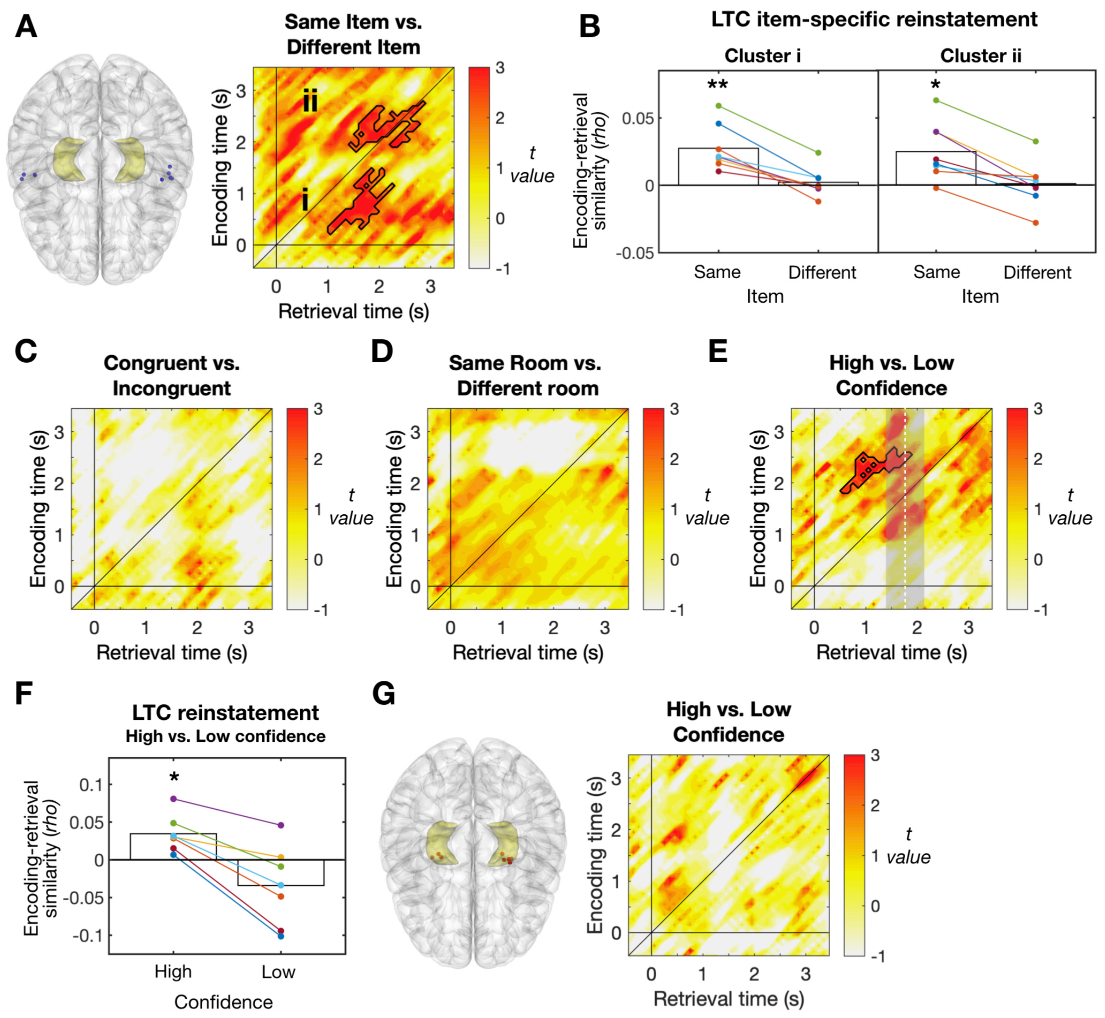

Supplementary Figure 19: LTC reinstatement in patients without HC epilepsy

(A) *T*-map contrasting encoding-retrieval similarity for same versus different items in the LTC. Areas of significant correlations are outlined in black. (B) Mean reinstatement in these clusters for "same item" and "different item" trials. (C) and (D) *T*-maps for congruent vs. incongruent and same vs. different contexts (rooms) contrasts. (E) Encoding-retrieval similarity in the "same item" condition for high and low confidence trials. Dashed line and shaded grey area shows mean response time ± S.E.M across the group of patients. (F) Reinstatement for high and low confidence trials in the cluster observed in E, tested against zero. (G) Absence of significant confidence effects in the hippocampus. *** and ** In panels B and F indicate *rho*-values that are across the group significantly different from zero at $p < 0.01$ and $p < 0.001$ respectively.

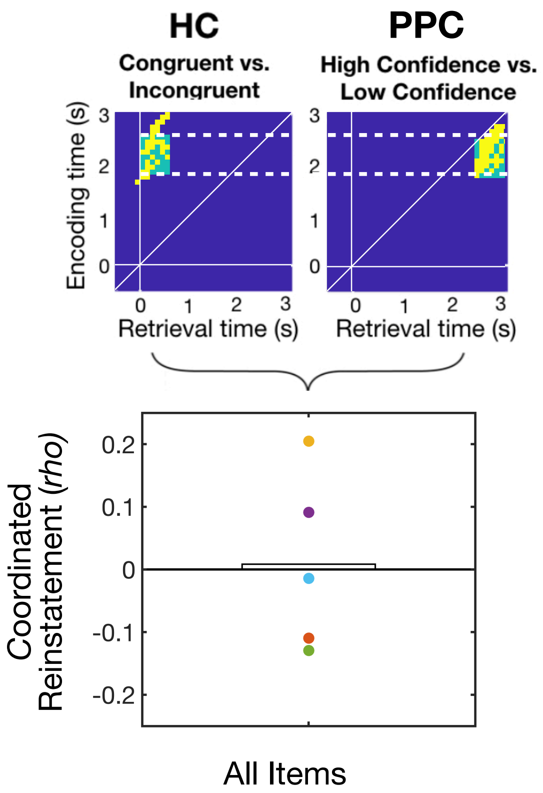

Supplementary Figure 20: Coordinated reinstatement between HC and PPC

Hippocampal and Posterior Parietal Cortex reinstatement is not correlated across trials.

Gender	Age	Risk factors	Medical history	Level of education	Age during first seizure	Seizure frequency	Aura	Semiology	Brain MRI	Seizure onset zone
M	26	Gestational diabetes	None	Secondary	16	1 per week	Deja vu, Autoscopic experience	Loss of consciousness, bilateral arm elevation	Normal	Right Occipital Fusiform
F	41	None	Twin sister with epilepsy	Primary	14	Several per day	Epigastric	Loss of consciousness, dystonic posturing of left arm, sTC	Bilateral dorsal parietal polymicrogyria	Left Precuneus Left Parietal Superior
M	23	Premature birth (25 weeks)	Spastic paralysis ADHD	Secondary	12	1 per month	Experience of levitation, cacosmia	Loss of consciousness, nystagmus, right OCD, dystonic posturing of right arm	Altered gyral pattern in bilateral precuneus.	Left Inferior Temporal Lobe
F	40	Low birth weight, febrile crisis	None	Tertiary	31	4 per month	Epigastric	Loss of consciousness, Rigth hemiface grimace	Normal	Left Amygdala
F	32	None	None	Secondary	17	4 per month	Deja vu	Loss of consciousness, OCD, sTC	Left middle temporal sulcus focal cortical dysplasia (FCD)	Left Middle Temporal Lobe
F	40	TCE at 12 years. Sister with	None	Tertiary	29	7 per month	Deja vu, ascending paresthesia	Language interruption, left hand desintonía, Loss	Left hippocampal atrophy	Left Temporal Pole
F	35	None	None	Tertiary	17	3-4 per week	Unspecific cephalic, palpitations	Loss of consciousness, oral automatisms, ictal speech	Extensive right temporal heterotopia	Right Anterior Hippocampus
M	46	None	None	Secondary	7	4 per day	Deja vu	Loss of consciousness, dystonic posturing of right arm , oral and hand automatisms, sTC	Focal cortical dysplasia (anterior left temporal)	Left Amygdala Left Anterior Hippocampus
M	49	mild TCE	None	Secondary	26	10-15 per month	Epigastric	Oral and bimanual automatisms	Possible amygdala dysplasia	Right Amygdala
M	30	TCE: Accident at 14 months	None	Tertiary	25	13 per month	Jamais vu	Global aphasia, Loss of consciousness	Encephalocele left temporal	Left Amygdala
M	23	None	AHS	Secondary	13	21 per month	Sadness	Drooling, bilateral arm dystonic posturing	Left hippocampal sclerosis (HS)	Left Anterior Hippocampus

TCE: Cranial Traumatism

ADHD (attention deficit and hyperactivity disorder)

AHS: Athletic heart syndrome

sTC = secondary tonic-clonic seizure

OCD: oculocephalic deviation

Supplementary Table 1: Information about patients and epilepsy types

SUPPLEMENTARY REFERENCES

1. Yaffe, R. B. *et al.* Reinstatement of distributed cortical oscillations occurs with precise spatiotemporal dynamics during successful memory retrieval. *Proc. Natl. Acad. Sci.* **111**, 18727–18732 (2014).
2. Eichenbaum, H. Memory: Organization and Control. *Annu. Rev. Psychol.* **68**, 19–45 (2017).
