# Supplementary material for: Coordinated representational reinstatement in the human hippocampus and lateral temporal cortex during episodic memory retrieval
Source: Nat Commun. 2019 May 21;10:2255. doi: 10.1038/s41467-019-09569-0 (PMC6529470; doi:10.1038/s41467-019-09569-0)
Supplement: Supplementary file 2 — Supplementary Data 1 [file 41467_2019_9569_MOESM2_ESM.docx]

| Subject | Electrodes | MNI Coordinates | | | |
| --- | --- | --- | --- | --- | --- |
| S1 | B1 | 34 | -13 | -23 |  |
|  | B2 | 37 | -13 | -22 |  |
|  | C1 | 33 | -31 | -10 |  |
|  | C2 | 37 | -31 | -10 |  |
|  | B8 | 60 | -9 | -19 |  |
|  | A9 | 55 | -1 | -21 |  |
|  | A11 | 63 | -1 | -19 |  |
|  | D7 | 51 | -12 | -39 |  |
|  | D8 | 55 | -13 | -39 |  |
|  | E7 | 50 | -22 | -21 |  |
|  | E9 | 58 | -22 | -20 |  |
|  | T6 | 42 | 1 | -43 |  |
|  | T8 | 49 | -1 | -40 |  |
|  | U5 | 60 | -27 | 4 |  |
|  | U6 | 64 | -28 | 4 |  |
|  | U7 | 67 | -28 | 6 |  |
| S2 | B'1 | -32 | -12 | -22 |  |
|  | B'7 | -57 | -19 | -19 |  |
|  | B'9 | -65 | -22 | -17 |  |
|  | C'8 | -52 | -39 | -19 |  |
|  | C'9 | -56 | -42 | -17 |  |
|  | C8 | 58 | -13 | -28 |  |
|  | C9 | 63 | -12 | -30 |  |
|  | S'5 | -24 | -41 | 71 |  |
|  | J'10 | -42 | -53 | 56 |  |
|  | J'11 | -46 | -53 | 57 |  |
|  | P'5 | -21 | -65 | 49 |  |
| S3 | B'1 | -29 | -12 | -18 |  |
|  | B'2 | -33 | -11 | -19 |  |
|  | C'1 | -31 | -34 | -4 |  |
|  | C'2 | -35 | -34 | -5 |  |
|  | A'7 | -46 | 4 | -33 |  |
|  | A'8 | -50 | 6 | -34 |  |
|  | B'7 | -51 | -8 | -26 |  |
|  | B'8 | -55 | -8 | -27 |  |
|  | I'11 | -45 | -66 | 29 |  |
|  | P'10 | -39 | -54 | 52 |  |
|  | U'9 | -41 | -66 | 54 |  |
| S4 | B'1 | -25 | -11 | -21 |  |
|  | B'2 | -29 | -10 | -20 |  |
|  | C'1 | -25 | -29 | -15 |  |
|  | C'2 | -30 | -29 | -13 |  |
|  | A'11 | -58 | 0 | -25 |  |
|  | A'12 | -62 | 0 | -24 |  |
|  | B'8 | -54 | -10 | -21 |  |
|  | B'9 | -58 | -10 | -21 |  |
|  | B'10 | -62 | -10 | -22 |  |
|  | C'9 | -58 | -24 | -11 |  |
|  | C'11 | -65 | -21 | -12 |  |
|  | C'12 | -68 | -18 | -12 |  |
|  | D'10 | -55 | -30 | -19 |  |
|  | D'12 | -63 | -29 | -19 |  |
|  | E'7 | -48 | -6 | -30 |  |
|  | E'8 | -52 | -6 | -30 |  |
|  | T'7 | -48 | 11 | -35 |  |
|  | T'8 | -52 | 12 | -35 |  |
| S5 | B'1 | -24 | -16 | -22 |  |
|  | B'2 | -28 | -15 | -22 |  |
|  | B'9 | -56 | -13 | -19 |  |
|  | A'8 | -46 | 4 | -25 |  |
|  | A'9 | -51 | 4 | -24 |  |
|  | A'10 | -55 | 5 | -25 |  |
|  | B'8 | -52 | -14 | -19 |  |
|  | B'10 | -60 | -11 | -19 |  |
|  | C'10 | -51 | -32 | -1 |  |
|  | C'11 | -54 | -30 | 0 |  |
|  | C'12 | -58 | -29 | 1 |  |
|  | D'11 | -60 | -31 | -15 |  |
|  | E'8 | -53 | -3 | -33 |  |
| S6 | B1 | 26 | -13 | -24 |  |
|  | B2 | 30 | -13 | -24 |  |
|  | C1 | 25 | -31 | -6 |  |
|  | C2 | 28 | -30 | -7 |  |
|  | B'8 | -49 | -8 | -19 |  |
|  | B'9 | -53 | -8 | -18 |  |
|  | B'10 | -57 | -8 | -17 |  |
|  | B'11 | -62 | -8 | -17 |  |
|  | D'11 | -60 | -16 | -19 |  |
|  | V'5 | -55 | 3 | -12 |  |
|  | V'6 | -58 | 3 | -10 |  |
|  | V'7 | -62 | 3 | -8 |  |
|  | W'9 | -66 | -32 | 8 |  |
|  | A8 | 47 | -2 | -23 |  |
|  | A9 | 51 | -2 | -24 |  |
|  | B8 | 54 | -12 | -23 |  |
|  | B9 | 58 | -12 | -22 |  |
|  | B10 | 62 | -11 | -23 |  |
|  | C12 | 66 | -28 | -15 |  |
| S7 | B1 | 28 | -11 | -25 |  |
|  | B2 | 32 | -10 | -24 |  |
|  | C1 | 23 | -22 | -17 |  |
|  | C2 | 28 | -22 | -17 |  |
|  | B9 | 58 | -12 | -18 |  |
|  | A9 | 54 | 0 | -26 |  |
|  | A10 | 58 | 1 | -25 |  |
|  | B10 | 61 | -12 | -17 |  |
|  | C12 | 66 | -25 | -18 |  |
|  | D9 | 54 | -12 | -30 |  |
|  | D10 | 57 | -12 | -30 |  |
|  | F10 | 59 | -31 | -11 |  |
|  | F11 | 63 | -31 | -11 |  |
|  | J8 | 55 | -42 | 15 |  |
|  | J9 | 58 | -43 | 16 |  |
|  | K9 | 63 | -53 | -9 |  |
|  | L8 | 60 | -43 | -3 |  |
|  | L9 | 64 | -43 | -2 |  |
|  | O8 | 60 | -64 | 1 |  |
|  | Q8 | 58 | -49 | 7 |  |
|  | Q9 | 62 | -50 | 8 |  |
|  | V10 | 42 | -61 | 17 |  |
|  | V11 | 46 | -61 | 18 |  |
|  | P11 | 43 | -38 | 41 |  |
|  | P12 | 47 | -39 | 42 |  |
|  | P13 | 51 | -39 | 42 |  |
|  | T10 | 56 | -37 | 36 |  |
|  | T11 | 59 | -37 | 39 |  |
|  | T12 | 62 | -37 | 40 |  |
|  | Y9 | 34 | -57 | 28 |  |
|  | Y10 | 34 | -59 | 32 |  |
| S8 | B'10 | -62 | -14 | -22 |  |
|  | A'9 | -53 | -5 | -28 |  |
|  | A'10 | -57 | -5 | -27 |  |
|  | B'8 | -54 | -14 | -23 |  |
|  | B'9 | -58 | -14 | -22 |  |
|  | U'3 | -53 | -16 | -2 |  |
|  | U'5 | -60 | -18 | -3 |  |
|  | J'15 | -54 | -48 | 31 |  |
|  | J'17 | -60 | -50 | 30 |  |
| S9 | B10 | 62 | -12 | -17 |  |
|  | A7 | 48 | -4 | -19 |  |
|  | A8 | 51 | -4 | -18 |  |
|  | A9 | 55 | -4 | -18 |  |
|  | B11 | 66 | -13 | -18 |  |
|  | B12 | 70 | -12 | -18 |  |
|  | C12 | 68 | -34 | -2 |  |
|  | E7 | 51 | -8 | -28 |  |
|  | E8 | 54 | -6 | -27 |  |
|  | E9 | 58 | -5 | -26 |  |
|  | S14 | 55 | -52 | 15 |  |
|  | S15 | 58 | -52 | 15 |  |
|  | T7 | 48 | 7 | -31 |  |
|  | T8 | 52 | 7 | -30 |  |
|  | T10 | 59 | 8 | -29 |  |
|  | Q14 | 56 | -51 | 33 |  |
| S10 | B'1 | -36 | -12 | -26 |  |
|  | B'2 | -42 | -13 | -29 |  |
|  | C'1 | -26 | 33 | -12 |  |
|  | B'6 | -67 | -13 | -33 |  |
|  | C'8 | -54 | -26 | -7 |  |
|  | C'9 | -58 | -24 | -6 |  |
|  | T'4 | -46 | 4 | -45 |  |
|  | T'5 | -52 | 2 | -46 |  |
|  | E'8 | -57 | -31 | -21 |  |
|  | E'9 | -62 | -30 | -18 |  |
|  | E'10 | -66 | -30 | -17 |  |
| S11 | A'8 | -46 | 28 | -5 |  |
|  | A'9 | -49 | 29 | -6 |  |
|  | B'7 | -44 | 11 | -3 |  |
|  | B'8 | -48 | 10 | -2 |  |
|  | B'9 | -51 | 11 | -1 |  |
|  | B'10 | -55 | 11 | 0 |  |

**Supplementary Data 1: Electrode locations in MNI space**

Table shows all electrodes in all patients. Hippocampal (red) and LTC (blue) electrodes included in the main analysis are indicated.
